# What factors drive or hinder drugstores and residents from jointly recycling expired drugs? An evolutionary game analysis

**DOI:** 10.3389/fpubh.2026.1777763

**Published:** 2026-03-05

**Authors:** Shiying Jiang, Chunyan Ma, Xiaochun Zeng

**Affiliations:** 1 School of Public Health, Hubei University of Medicine, Shiyan, China; 2 School of Electrical Engineering, Hubei University of Automotive Technology, Shiyan, China

**Keywords:** driving factors, evolutionary game, expired drugs, hindering factors, joint recycling drugs

## Abstract

**Background:**

Expired drugs are widely present in various countries and regions around the world and have serious adverse effects on land, the natural environment, public health, and economic development. Therefore, it is important to explore how drugstores can collaborate with residents to recycle these drugs. However, there is currently a lack of systematic research in this field.

**Methods:**

To fill this research gap, this article applies evolutionary game theory to establish a theoretical model.

**Results:**

The cooperative evolution trend and stable strategies between the two parties are analyzed, and the driving and hindering factors are also explored. Numerical simulation and sensitivity analysis indicate that socioeconomic performance and moderate reward outputs are key driving factors influencing joint recycling behavior between drugstores and residents. In contrast, recycling costs, time loss, promotional costs, large rewards, and small rewards are hindering factors. This article also identifies the value ranges for these factors.

**Conclusion:**

The proposed model and results have theoretical significance for drugstore recycling strategies and resident participation, and they provide a reference for ecological environment protection and the development of the drug recycling market.

## 1 Introduction

Unused and expired drugs are widely present in households around the world, posing significant adverse effects on the land environment (such as animals, water, and plants), public health, and the economy (1–3). Some illegal companies collect these drugs through informal channels and then conduct secondary processing or direct sales, which not only disrupt the normal market environment but also pose a great threat and risk to residents' health (4, 5). In 2024, the “Focus Interview” program of China Central Television exposed a case involving the illegal recycling and resale of drugs worth up to 200 million yuan (6). In fact, some drugstores in China have initiated the recycling program for expired drugs, but the results have not been satisfactory (7). Many residents are unclear about scientific disposal methods, lack knowledge of proper drug handling, and show weak willingness to participate in recycling programs (8, 9). Therefore, exploring how drugstores and residents can effectively cooperate to jointly recycle these drugs, thereby reducing the harm to the natural environment and society, has become a highly meaningful research topic.

Therefore, it can be concluded that residents rarely hand over unused and expired drugs to professional institutions for recycling and scientific disposal. This raises several important questions: Why is the enthusiasm of residents for participating in drug recycling low? What are the reasons? How can drugstores collaborate dynamically with residents to recycle a greater quantity of expired drugs?

To answer these research questions, we constructed an evolutionary game model to analyze the behavior of drugstores and residents. We considered both driving and hindering factors. The cooperative evolution trend and stable strategy of the two parties were analyzed. Numerical simulation and sensitivity analysis were conducted to support the conclusions.

To the best of the author's knowledge, existing studies have not analyzed the cooperative relationship between drugstores and residents based on evolutionary game theory, nor have they systematically examined the driving factors and obstacles influencing cooperation between the two parties. Healthcare providers play a vital role in improving medication management and reducing waste (10). A national cross-sectional online survey was conducted among 1,596 pharmacists, and it indicated that drugstore pharmacists play a crucial role in the recycling of expired drugs, highlighting a pressing need for continuous training for pharmacists (11). Therefore, the significant role of drugstores in drug recycling warrants further research. This study makes the following original contributions:

(1) An evolutionary game model for drug recycling by drugstores and residents is proposed. This model overcomes the limitations of the majority of previous studies, which focuses only on residents, by treating drugstores as stakeholders. The numerical simulation shows that the participation of drugstores in drug recycling can reach a stable state and that it is feasible.
(2) This study theoretically demonstrates that socioeconomic performance and moderate reward outputs are driving factors for the joint recycling of expired drugs, whereas large reward and small reward outputs are hindering factors.
(3) The analysis results have practical reference value for the government and related industries in formulating relevant policies, establishing industry standards, and promoting the development of the expired drug recycling market.

The remainder of this article is organized as follows. In Section 2, we present the literature review. In Section 3, we provide a detailed description of the theoretical hypothesis, the model construction process, and the robustness tests of the proposed model. In Section 4, the equilibrium analysis of the evolutionary game is investigated. In Section 5, the influence of some key factors on the willingness to cooperate in drug recycling is examined using the numerical simulation method, and the results are presented. In addition, a sensitivity analysis of the factors is conducted. Section 6 concludes the article, proposes some suggestions for the development of the drug recycling industry, and suggests future research directions.

## 2 Literature review

Some scholars have analyzed residents' knowledge, attitudes, disposal methods, and willingness to participate in the recycling of unused and expired drugs. Alssageer et al. (12) reported that more than three-fourths of participants stated that they were never taught to properly handle medications, and 60.8% considered the medical team to be the best source of such knowledge. A literature review on current practices and contamination risks associated with pharmaceutical disposal in low-income settings in Africa was conducted. The results showed that unused and expired drugs were disposed of in pit latrines and with household solid waste (13). Based on a survey of residents (14), 62.2% reported donating their unused drugs. In terms of disposal methods, 63.5%, 55.2%, and 44.8% of participants discarded expired drugs in bins or garbage, rinsed them in toilets or sinks, or burned them, respectively.

Unlike previous studies that focused on the general population, some scholars have examined healthcare personnel. To evaluate attitudes and practices toward unused and expired drugs in Dhaka, a study (15) found that 42.86% of nurses expressed concerns about immune system deficiencies caused by improper drug handling, and 69.79% of doctors disposed of drugs by throwing them into toilets, washbasins, or trash cans. Using a cross-sectional analysis, a study (16) pointed out that, due to tight schedules, the majority of doctors and nurses used unsafe methods for handling expired and excess drugs. Therefore, the knowledge of healthcare professionals regarding expired drugs is limited, and the scientific rigor of their handling practices needs to be further enhanced.

Some scholars have further explored the factors influencing residents' participation in drug recycling. These factors can be grouped into three main categories: Individual, government, and supporting environmental factors. Regarding individual factors, personal education, knowledge and experience, age, occupation, attitude, and health awareness are the most commonly cited influences. Tegegne et al. (17) pointed out that receiving information, having taken medication in the past 6 months, and being aware of the situation are key factors influencing the public's positive attitude toward the disposal of unused drugs. A study using multivariate logistic regression analysis identified key factors associated with drug storage: Housewives and government employees are less likely to store unused drugs compared to students and workers (10). Srijuntrapun et al. (18) analyzed 400 families in Thailand and concluded that age, education, attitudes, and perception can better influence the correct handling of unused or expired drugs. Lv et al. constructed a chain mediation model. Using a sample size of 366 residents, the study found that personal health awareness moderates the chain mediation path by strengthening the positive effect of return intention on proper return behavior (19).

The government plays an important role in drug recycling by providing subsidies, enacting legislation, and conducting supervision. Huang et al. suggested that the government should design a subsidy policy to encourage users to return their expired drugs. They established a tri-level programming model to study how the government could optimize an expired drug recycling logistics network and determine appropriate subsidy policies (20). Sabrina concluded that the US has implemented various drug recycling or redistribution programs for an estimated 3–7% of unused pharmaceutical products, while Canada has made only limited efforts in drug recycling and faces numerous regulatory and legislative obstacles to establishing such programs. Therefore, strengthening legislation and regulation is of great importance (21).

In terms of creating a supportive environment for the recycling of expired drugs, measures such as introducing information systems, establishing collection points, and developing a recycling system can enhance convenience and facilitate the smooth implementation of recycling activities. Luo and Reimers suggested that by constructing an information infrastructure that connects waste drug recycling, medication management, and household drug management practices, the incentives for all involved actors to participate in the integrated drug recycling system could be dramatically increased (22). After conducting a questionnaire survey, Mahara et al. pointed out that to improve the resident's awareness about family expired drug disposal and to ensure financial support for the recycling process, it is important to establish an accessible and convenient recycling point and introduce relevant laws and regulations to support a long-term recycling mechanism (23). Kksoy collected data in Burdur, Türkiye, using a non-probability sampling technique, and found that medicines are not disposed of properly, with some expiring eventually. There is a need for public awareness regarding this issue. A “drug take-back system” for unused medicines can be useful in solving this problem (24).

Based on the literature reviewed above, there are several gaps. Few studies have examined how drugstores and residents can jointly engage in recycling. There is no clear role model, and there is a lack of theoretical understanding of the dynamic process of expired drug recycling between drugstores and residents. Moreover, the driving and hindering factors influencing expired drug recycling warrant further analysis.

The reason for choosing to build an evolutionary game model is based on the research problem. Evolutionary game theory has been extensively applied in various waste recycling fields, such as abandoned shared bicycles (25), photovoltaic recycling (26), power battery recycling (27), and cross-regional cooperation in waste recycling (28). This study not only focuses on the equilibrium strategies but also pays attention to the dynamic evolution process of stakeholders' decision-making and changes in the willingness of both parties to cooperate. The dynamic process, uncertainty, and group behavior emphasized by evolutionary game theory are sufficient to prove the rationality for analyzing behavioral strategies of drugstores and residents using an evolutionary game model. The core of evolutionary game analysis is not to predict the optimal strategy results of one-time selection, but to focus more on the strategy adjustment process, trend and stability of limited rational groups in the long-term selection process (25, 27). Therefore, we have chosen evolutionary game theory as the theoretical tool.

## 3 Evolutionary game theory among drugstores and residents

This section presents the problem description and constructs the replicator dynamic equation using evolutionary game theory.

### 3.1 Problem description and basic assumptions

Drugstores often inform residents about the hazards of expired drugs and provide guidance on how to handle them. Under the influence of drugstore publicity, residents are willing to actively participate in drug recycling. Residents collect expired and excess drugs from their homes, store them appropriately, and then regularly or irregularly deliver them to drugstores, which takes time *t*. In different recycling scenarios, the time spent by residents is different, which is denoted as *t*_1_ *and t*_2_ (2, 29). After rationalizing the collected drugs, residents receive certain rewards from drugstores, such as eggs, chewing gum, beverages, small discount coupons, and medicine vouchers. These rewards are denoted as *m* (11, 30). Residents gain personal value by actively participating in drug recycling activities and enhancing their awareness of environmental protection, which encourages them to accept low-value rewards (8, 19, 31). During the process of collecting expired drugs, drugstores also invest significant manpower and time, and the resulting costs are denoted as *c*_2_ (32–34). As mentioned earlier, drugstores actively inform residents about the hazards of expired drugs, which incurs a promotional cost *c*_1_. It can be seen that, in the process of drug recycling, drugstores also engage in significant public welfare activities, thereby enhancing their social image. This, in turn, attracts more residents to purchase drugs, potentially increasing profits (10, 11). The resulting socioeconomic performance is denoted as *s*.

Drugstores and residents engage in a cooperative game to jointly recycle drugs. At present, in the field of expired drug recycling, the government rarely implements incentive policies to guide enterprise behavior, so we only consider the scenario without government intervention here. There are four types of cooperative scenarios: (1,1), (1,0), (0,1), and (0,0) (35–37). The probability that drugstores participate in drug recycling is denoted as ϕ, and the probability that residents participate is denoted as Ω. Residents' time costs may vary across different collaborative scenarios (38, 39). In scenario (1,1), residents spend time participating in recycling, and they receive rewards from drugstores, so the payoff of residents is *m*−*t*_1_. Drugstores gain a good reputation for recycling drugs, but at the same time, they have to publicize and bear the cost of recycling, so the payoff is *sc*_1_*c*_2_*m*. In scenario (1,0), drugstores engage in publicity and therefore incur a cost of *c*_1_. Residents do not participate in drug recycling, so the payoff is 0. In scenario (0,1), because drugstores are not prepared to participate in drug recycling, the payoff is 0. Although residents want to participate and it takes a certain amount of time *t*_2_, in the end, recycling cannot be completed, and the payoff is −*t*_2_. In scenario (0,0), drugstores and residents are not involved in drug recycling, so the payoff is 0.

According to these basic assumptions, we can obtain a payoff matrix for drugstores and residents, as shown in Table 1.

**Table 1:** Payoff matrix.

		**Drugstores**	
		**Drugs recycling** **(ϕ)**	**No recycling** **(1−ϕ)**
Residents	Participating in recycling (Ω)	*m*−*t*_1_, *s*−*c*_1_−*c*_2_−*m*	−*t*_2_, 0
	Not participating in recycling (1−Ω)	0, −*c*_1_	0, 0

### 3.2 The replicator dynamic equation for participants

The replicator dynamic equations for each of the two parties can now be established. For drugstores, the expected utility value from participating in drug recycling and from not participating in drug recycling is denoted by ξ_*d*1_ and ξ_*d*2_, respectively. Therefore, we have the following equations:


$$\begin{array}{l} \xi_{d1}=\phi \left(s-c_{1}{-c}_{2}-m\right)-\left(1-\phi \right)c_{1} \end{array}$$ (1)

$$\begin{array}{l} \xi_{d2}=0 \end{array}$$ (2)

The average expected utility for drugstores is calculated as:


$$\begin{array}{l} \bar{\xi_{d}}=\varphi \xi_{d1}+\left(1-\phi \right)\xi_{d2\;} \end{array}$$ (3)

The replicator dynamic equation is a differential equation that represents the growth rate of the probability over time. Thus, the replicator dynamic equation for drugstores can be expressed as:


$$\begin{array}{l} \epsilon \left(\phi \right)=\frac{d\phi }{dt}=\phi \left(1-\phi \right)\left\{\Omega \left(s{-c}_{2}-m\right)-c_{1}\right\} \end{array}$$ (4)

For residents, assuming that the expected utility of participating in recycling is ϑ_*c*1_, we can develop a replicator dynamic equation, where ϑ_*c*1_ can be written as:


$$\begin{array}{l} \vartheta_{c1}=\phi \left(m-t_{1}\right)+\left(1-\phi \right)\left(-t_{2}\right) \end{array}$$ (5)

The expected utility of not participating in recycling, ϑ_*c*2_, is expressed as:


$$\begin{array}{l} \vartheta_{c2}=0\; \end{array}$$ (6)

The average expected utility for residents is calculated as:


$$\begin{array}{l} \bar{\vartheta_{c}}=\Omega \vartheta_{c1}+\left(1-\Omega \right)\vartheta_{c2} \end{array}$$ (7)

Thus, the replicator dynamic equation for residents is as follows:


$$\begin{array}{l} \delta \left(\Omega \right)=\frac{d\Omega }{dt}=\Omega \left(1-\Omega \right)\left\{\phi \left(m-t_{1}+t_{2}\right)-t_{2}\right\} \end{array}$$ (8)

By combining Equations 7, 8 the dynamic equations for the two-party game can be expressed as:


$$\{\begin{array}{c} \epsilon (\phi )=\phi (1-\phi )\{\Omega (s-c_{2}-m)-c_{1}\}\\ \delta (\Omega )=\Omega (1-\Omega )\{\phi (m-t_{1}+t_{2})-t_{2}\} \end{array}$$ (9)

According to the simultaneous dynamic equations, simultaneously using ϵ(ϕ) = 0 and δ(Ω) = 0

To solve the equations, there are five local equilibrium points *E*_1_(1, 1), *E*_2_(0, 1), *E*_3_(0, 0), *E*_4_(1, 0), $E_{5}\left(\phi^{*},\Omega^{*}\right)$, where $E_{5}\left(\phi^{*},\Omega^{*}\right)$ satisfies the following simultaneous equations:


$$\{\begin{array}{c} (1-\phi )\{\Omega (s-c_{2}-m)-c_{1}\}=0\\ (1-\Omega )\{\phi (m-t_{1}+t_{2})-t_{2}\}=0 \end{array}$$ (10)

## 4 Stability analysis of the evolutionary game model

Based on the previous replicator dynamic equations, an analysis of the Jacobian matrix is conducted, and the evolutionary stable strategies are also analyzed. The goal of this article is to identify the evolutionary stable strategy of the two-party evolutionary game among the equilibrium points. Friedman pointed out that ESS only appears in pure strategies (40). Therefore, this article examines the stability of four pure strategies in the two-party evolutionary game. The stability of the equilibrium points in a multi-group dynamic system can be determined using Lyapunov stability theory (41). According to Lyapunov stability theory, the stability of the equilibrium points can be further assessed through a local stability analysis of the Jacobian matrix. According to the replicator dynamic equation of each stakeholder, the Jacobian matrix J can be derived and expressed as:


$$\begin{array}{l} J=\left|\begin{array}{cc} \frac{d\epsilon (\phi )}{d\phi } & \frac{d\epsilon (\phi )}{d\Omega }\\ \frac{d\delta (\Omega )}{d\phi } & \frac{d\delta (\Omega )}{d\Omega } \end{array}\right|\\ =\left|\begin{array}{cc} \left(1-2\phi )\{\Omega (s-c_{2}-m)-c_{1}\right\} & \phi (1-\phi )(s-c_{2}-m)\\ \Omega (1-\Omega )(m-t_{1}+t_{2}) & \left(1-2\Omega )\{\phi (m-t_{1}+t_{2})-t_{2}\right\} \end{array}\right| \end{array}$$ (11)

It is necessary to discuss the stability of the four local equilibrium points because they are not necessarily the evolutionary stability points of the replicator dynamic system. The system achieves local stability when *Det*(*J*)>0 *and Tr*(*J*) < 0 are satisfied simultaneously (42). According to the Jacobian matrix J, *Det*(*J*) and *Tr*(*J*) can be calculated, as shown in Table 2.

**Table 2:** Stable points.

**Equilibrium points**	**Det(J)**	**Tr(J)**	**Stability**	**Stable conditions**
*E*_1_(1, 1)	(*s*−*c*_1_−*c*_2_−*m*)(*m*−*t*_1_)	−(*s*−*c*_1_−*c*_2_−*m*)−(*m*−*t*_1_)	ESS	(*s*−*c*_1_−*c*_2_−*m*)(*m*−*t*_1_)>0,*t*_1_−*m*−*s*+*c*_1_+*c*_2_ < 0
*E*_2_(0, 1)	(*s*−*c*_1_−*c*_2_−*m*)*t*_2_	*s*−*c*_1_−*c*_2_−*m*+*t*_2_	ESS	(*s*−*c*_1_−*c*_2_−*m*)*t*_2_>0, *s*+_*t*_2_−*c*2_−*c*_1_ < 0
*E*_3_(0, 0)	*t*_2_(−*c*_1_)	*t*_2_−*c*_1_	ESS	*t*_2_−*c*_1_ < 0
*E*_4_(1, 0)	*c*_1_(*m*−*t*_1_)	*m*+*c*_1_−*t*_1_	ESS	*m*>*t*_1_,*m*+*c*_1_−*t*_1_ < 0
$E_{5}\left(\phi^{*},\Omega^{*}\right)$	(*s*−*c*_2_−*m*)(*m*−*t*_1_+*t*_2_)	*0*	NO	Local equilibrium

Based on the above analysis, it is sufficient to consider only the stability of the pure-strategy equilibrium. The system has four stable points: *E*_1_(1, 1), *E*_2_(0, 1), *E*_3_(0, 0), and *E*_4_(1, 0).

## 5 Sensitivity analysis and discussion

In this section, the key parameters of the model are specified based on the actual development context, and the model constructed in the previous section is simulated to analyze the system's evolutionary outcomes under different scenarios. The influence and sensitivity of some key factors on the evolutionary game outcomes between drugstores and residents are also investigated.

### 5.1 Initial parameters

For the sake of generality, the initial value of ϕ and Ω is set to 0.5. In this study, most of the assumptions are formulated or calculated according to the current conditions of the Chinese recycling industry. In addition, some parameter settings mainly refer to previously published studies. In addition, we interviewed several stakeholders, including some residents and drugstores. At present, the rewards offered by drugstores are relatively low. The initial parameters used in the evolutionary game analysis of this study are as follows: *s* = 670, *c*_1_ = 120, *c*_2_ = 260, *t*_1_ = 46,*t*_2_ = 15, *m* = 80. To intuitively observe the dynamic evolution of stakeholders' strategies, we simulate the dynamic evolution trajectories of stakeholders‘ strategies using MATLAB 2023.

### 5.2 ESS in the two-party game

**Figure 1:**
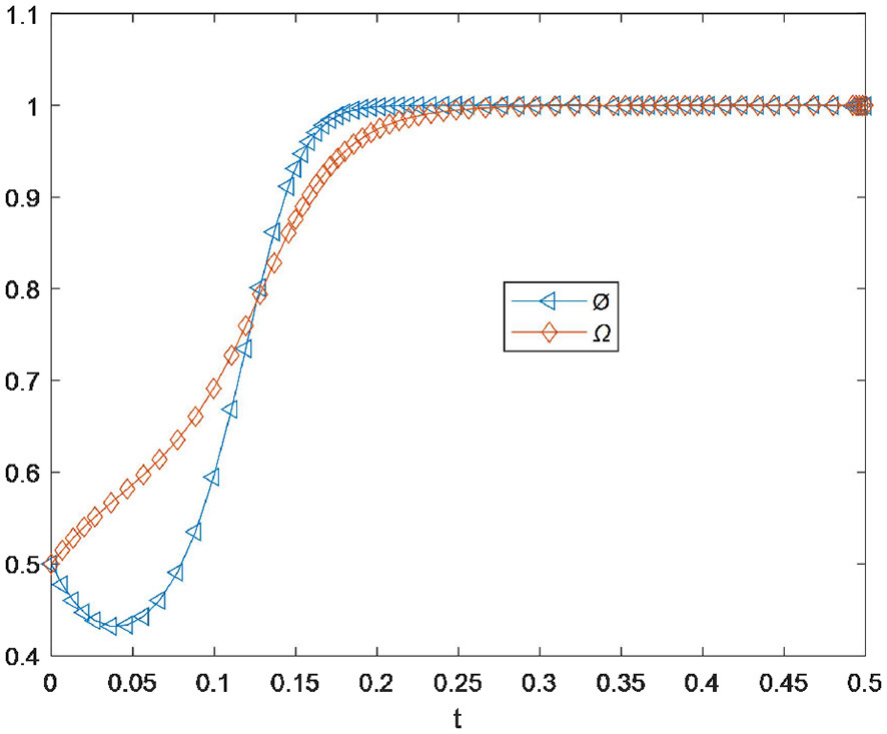
Stable state of *E*_1_(1, 1).

Before analyzing the influence of several parameters, we first examine whether the replicator dynamic equation can reach the stable state of *E*_1_(1, 1). As shown in Figure 1, residents and drugstores can actively choose to cooperate and reach *E*_1_(1, 1). Careful observation reveals that the willingness of residents to participate increases rapidly, while the willingness of drugstores to participate in the recycling process initially drops briefly below 0.4, then rebounds and reaches its maximum value at the time point of 0.20. Subsequently, the cooperation willingness curves of drugstores and residents become a coincident horizontal line. This indicates that drugstores can obtain greater social and economic benefits during the drug recycling process, while residents' participation is stimulated by the rewards provided by the drugstores (16, 29). Ultimately, both drugstores and residents are able to engage in dynamic cooperation effectively and jointly recycle expired drugs, achieving *E*_1_(1, 1).

### 5.3 Sensitivity analysis

Next, we analyze the impact of minor changes in individual factors on the evolutionary game outcomes between the two parties.

1) Socioeconomic performance *s*

**Figure 2:**
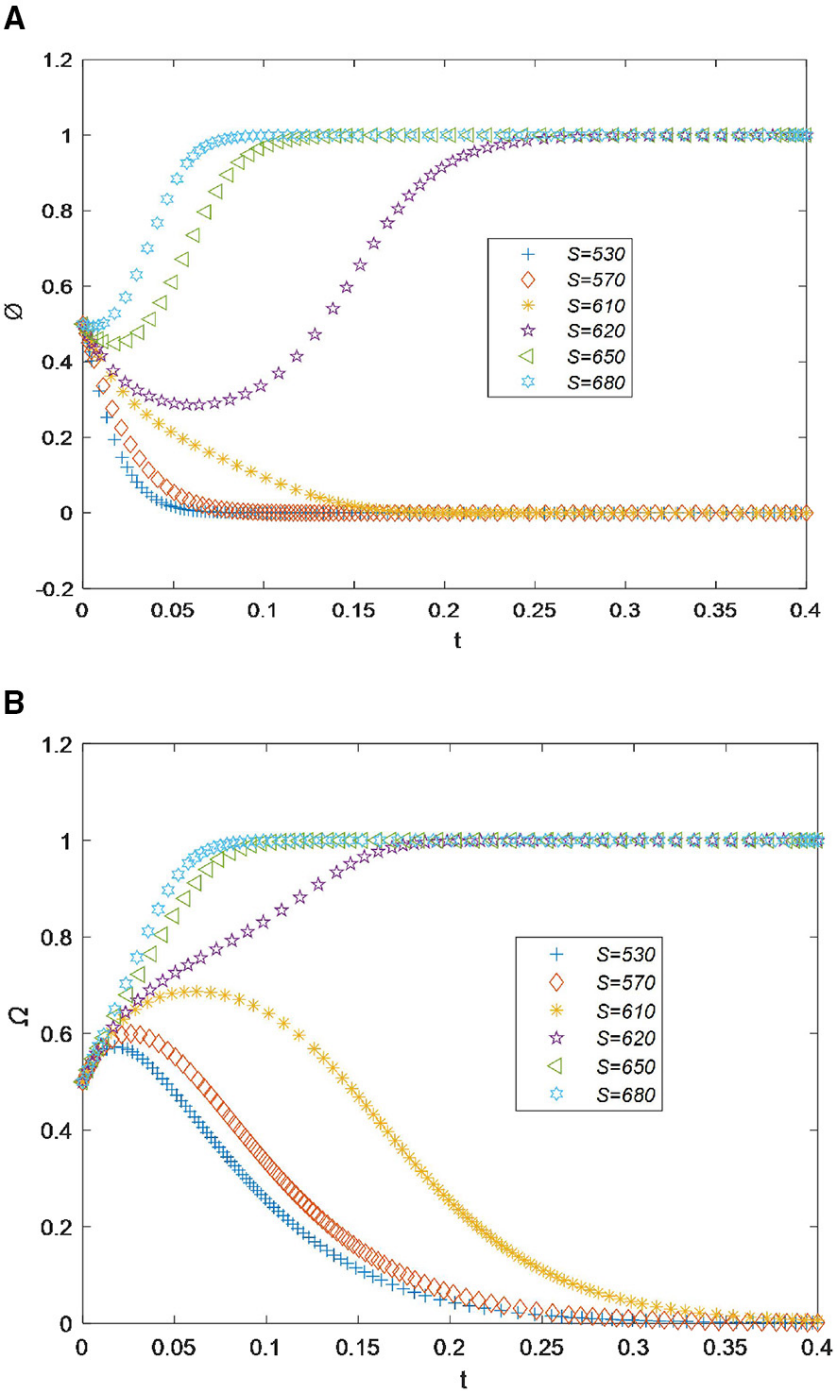
**(A)** Sensitivity analysis of socioeconomic performance for drugstores. **(B)** Sensitivity analysis of socioeconomic performance for residents.

In Figure 2A, it can be seen that when socioeconomic performance *s* is less than 610, the willingness of drugstores is extremely low, and it will rapidly decrease and approach 0 before the time point 0.15. This indicates that when socioeconomic performance is low, drugstores believe that participating in the drug recycling process would impose high economic and time costs, so they choose not to recycle drugs (43). However, when *s* increases by just 10, which is equal to 620, the recycling willingness curve of drugstores initially decreases slightly but then rapidly rises to 1.0. As *s* increases further, the recycling willingness reaches its maximum value of 1.0 before the time point 0.1, indicating a state of full cooperation in drug recycling. Overall, as socioeconomic performance increases, drugstores' willingness to recycle drugs will also gradually strengthen.

For residents, as shown in Figure 2B, when *s* is less than 610, the willingness curve to recycle drugs initially rises slightly and then drops rapidly. However, when *s* increases by only 10, the willingness value rapidly increases from 0.0 to 1.0 at the time point 0.20 and then remains stable. Therefore, based on the results shown in Figures 2A, B, it can be concluded that drugstores and residents are willing to cooperate and jointly recycle drugs only when socioeconomic performance exceeds 620. Drugstores have a dual role in social development. On the one hand, they contribute economically by promoting development. On the other hand, they serve a public welfare function by protecting residents' health. Therefore, when drugstores gain socioeconomic benefits from recycling expired drugs, their enthusiasm for participation is stimulated. They will actively provide convenience measures and incentives to encourage residents to participate in recycling. As a result, they reach an optimal state of cooperation.

2) Promotional cost

**Figure 3:**
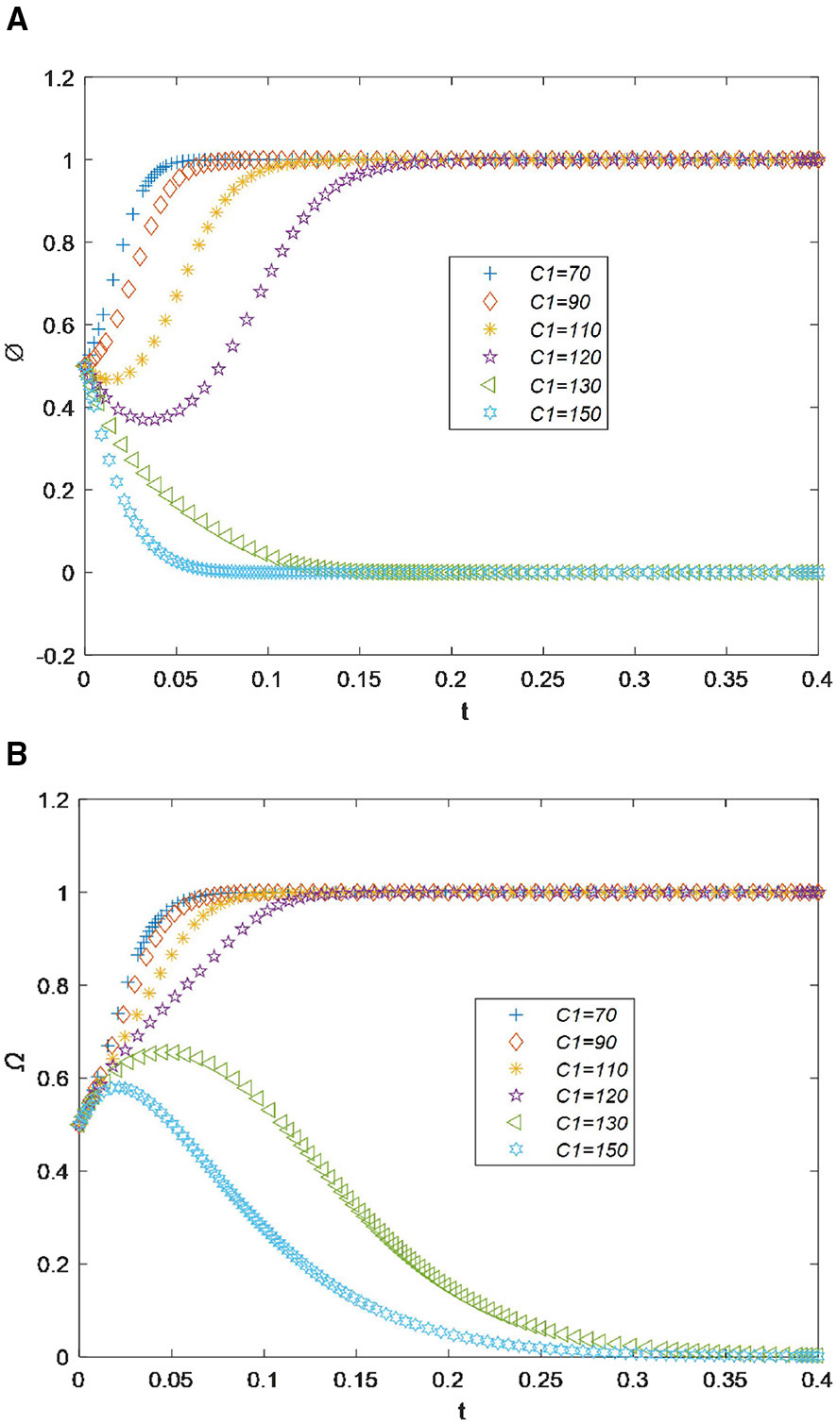
**(A)** Sensitivity analysis of the promotional cost for drugstores. **(B)** Sensitivity analysis of the promotional cost for residents.

In Figure 3A, we can see that when the promotional cost *c*_1_ is less than 120, the willingness of drugstores to recycle drugs reaches its maximum value very quickly, before the time point of 0.15. This indicates that lower promotional costs do not affect the drug recycling behavior of drugstores (23). However, when *c*_1_ increases by only 10, the recycling willingness value drops to 0 at the time point of 0.12. This suggests that the cost has become too high and exceeds drugstores' tolerance threshold. Subsequently, as *c*_1_ increases further, the recycling willingness curve drops sharply and eventually approaches 0. Overall, as *c*_1_ increases, the willingness of drugstores to recycle drugs decreases.

For residents, as shown in Figure 3B, the willingness to recycle drugs reaches its maximum value before the time point of 0.15. However, when the cost *c*_1_ increases slightly from 120 to 130, the recycling willingness value drops sharply over time and approaches 0 by the time point of 0.3. Therefore, based on the results shown in Figures 3A, B, we can draw the conclusion that drugstores and residents are willing to cooperate and jointly recycle drugs only when the promotional cost is less than 120,.

3) Recycling cost

**Figure 4:**
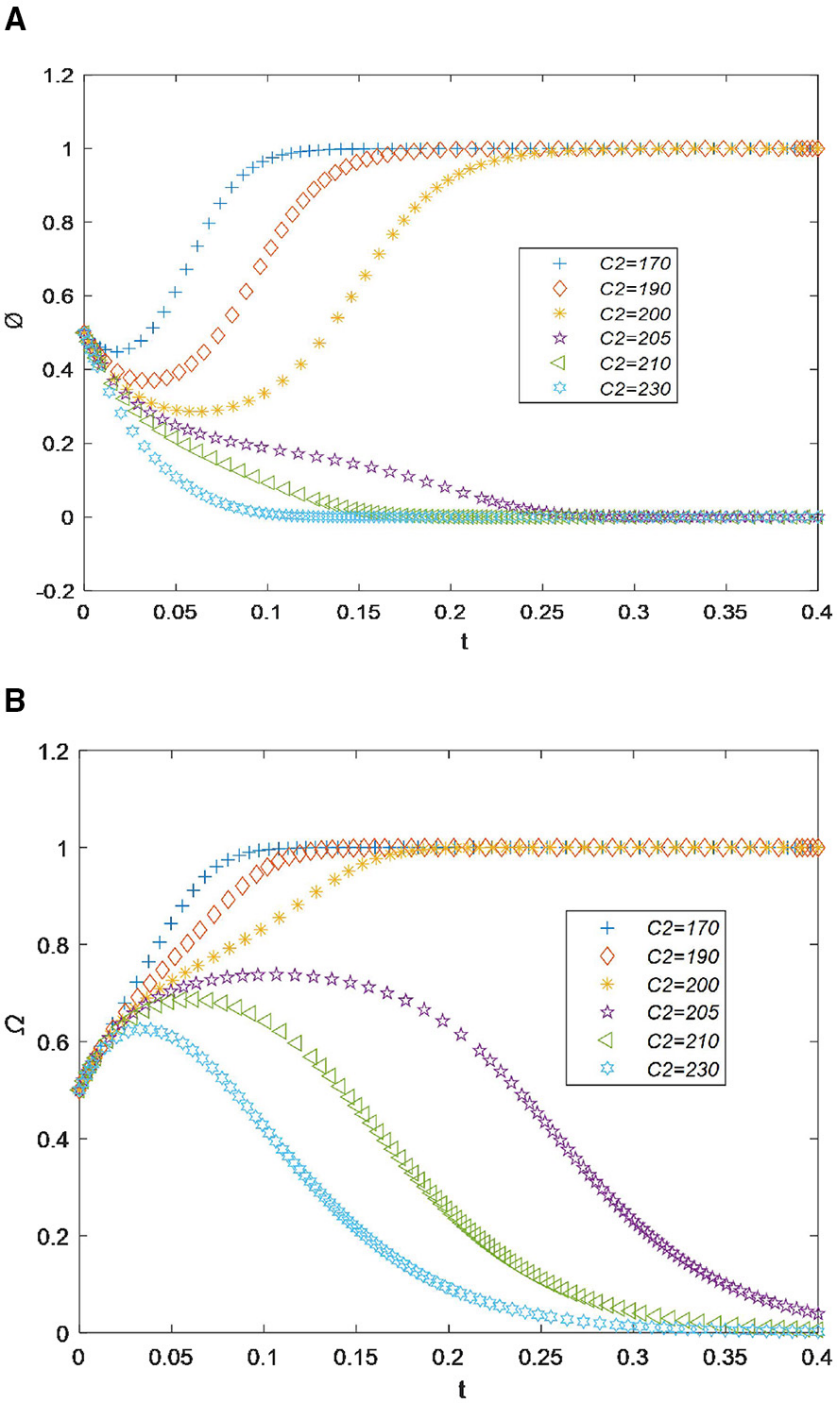
**(A)** Sensitivity analysis of the recycling cost for drugstores. **(B)** Sensitivity analysis of the recycling cost for residents.

In Figure 4A, it can be seen that when the recycling cost *c*_2_ is less than 200, the willingness of drugstores to recycle drugs reaches its maximum value very quickly, before the time point of 0.25. This indicates that lower costs do not affect the drug recycling behavior of drugstores (18). However, when *c*_2_ increases by only 10, the recycling willingness value drops to 0 at the time point of 0.15. This suggests that the cost has become too high and exceeds drugstores' tolerance threshold. Subsequently, as *c*_2_ increases further, the recycling willingness curve drops sharply and eventually approaches 0. Overall, as *c*_1_ increases, the willingness of drugstores to recycle drugs decreases.

For residents, as shown in Figure 4B, the willingness to recycle drugs reaches its maximum value before the time point of 0.20. However, when the cost *c*_2_ increases from 200 to 210, the recycling willingness value quickly drops to the minimum value of 0 after a slight increase at the time point of 0.30. Therefore, based on the results shown in Figures 4A, B, we can draw the conclusion that drugstores and residents are willing to cooperate and jointly recycle drugs only when the recycling cost is less than 200.

4) Time loss

**Figure 5:**
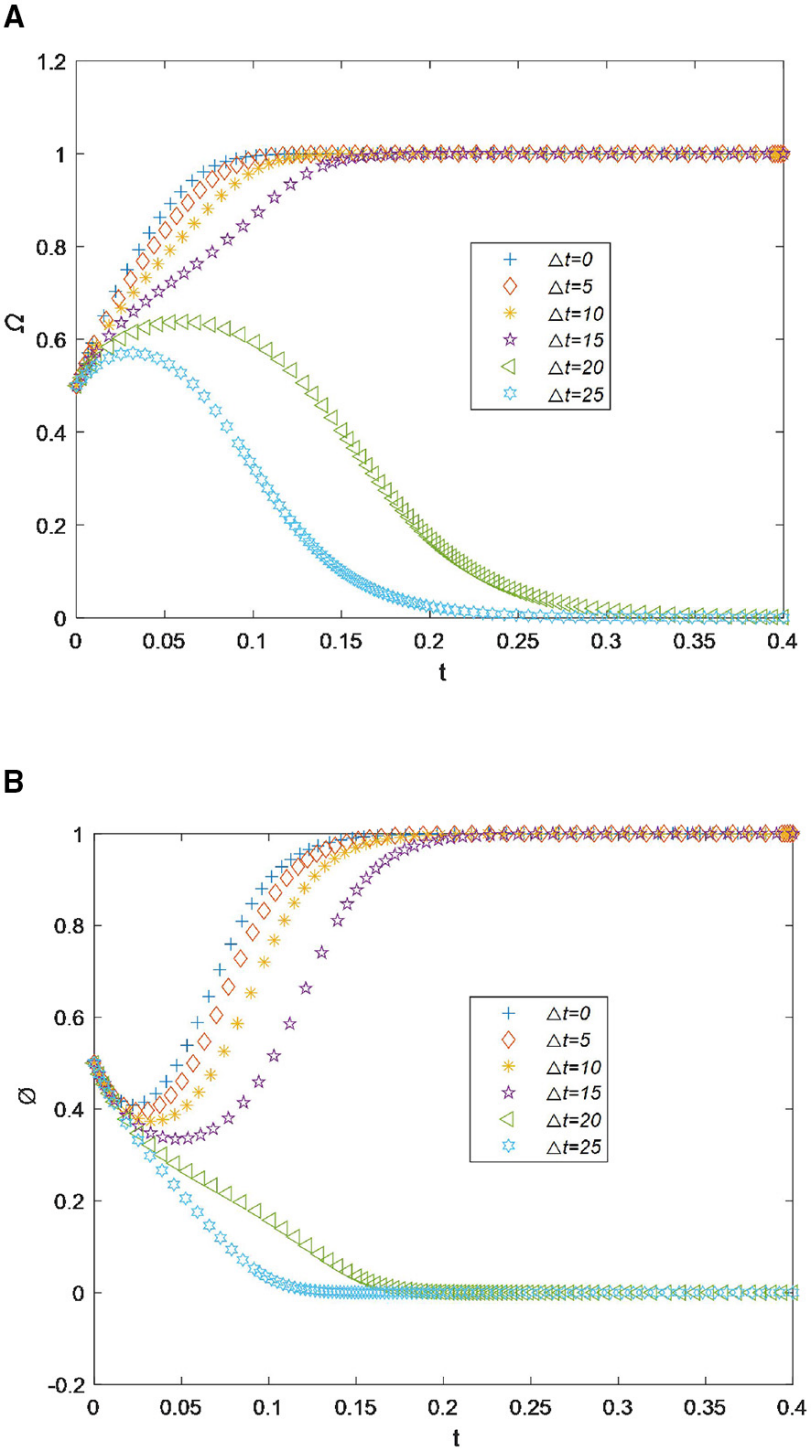
**(A)** Sensitivity analysis of time loss for drugstores. **(B)** Sensitivity analysis of time loss for residents.

For drugstores, as shown in Figure 5A, their willingness to recycle drugs reaches its maximum value before the time point of 0.20. However, when the time loss *t* increases slightly from 70 to 75, the recycling willingness value quickly drops to the minimum value of 0 at the time point of 0.18. Overall, as *t* increases, the willingness of drugstores to recycle drugs decreases.

In Figure 5B, it can be seen that when the time loss *t* is less than 70, residents' willingness to recycle drugs reaches its maximum value very quickly, before the time point of 0.20. This indicates that a small time loss does not affect the drug recycling behavior of residents. However, when *t* increases by only 5, the recycling willingness value quickly drops to the minimum value of 0 after a slight increase at the time point of 0.30. This phenomenon indicates that residents have other, more important matters to deal with and are unwilling to spend significant time collecting expired drugs (8, 44). Subsequently, as *t* increases further, the recycling willingness curve drops sharply and eventually approaches 0. Therefore, based on the results shown in Figures 5A, B, we can draw the conclusion that drugstores and residents are willing to cooperate and jointly recycle drugs only when the time loss is less than 70.

5) Reward output

**Figure 6:**
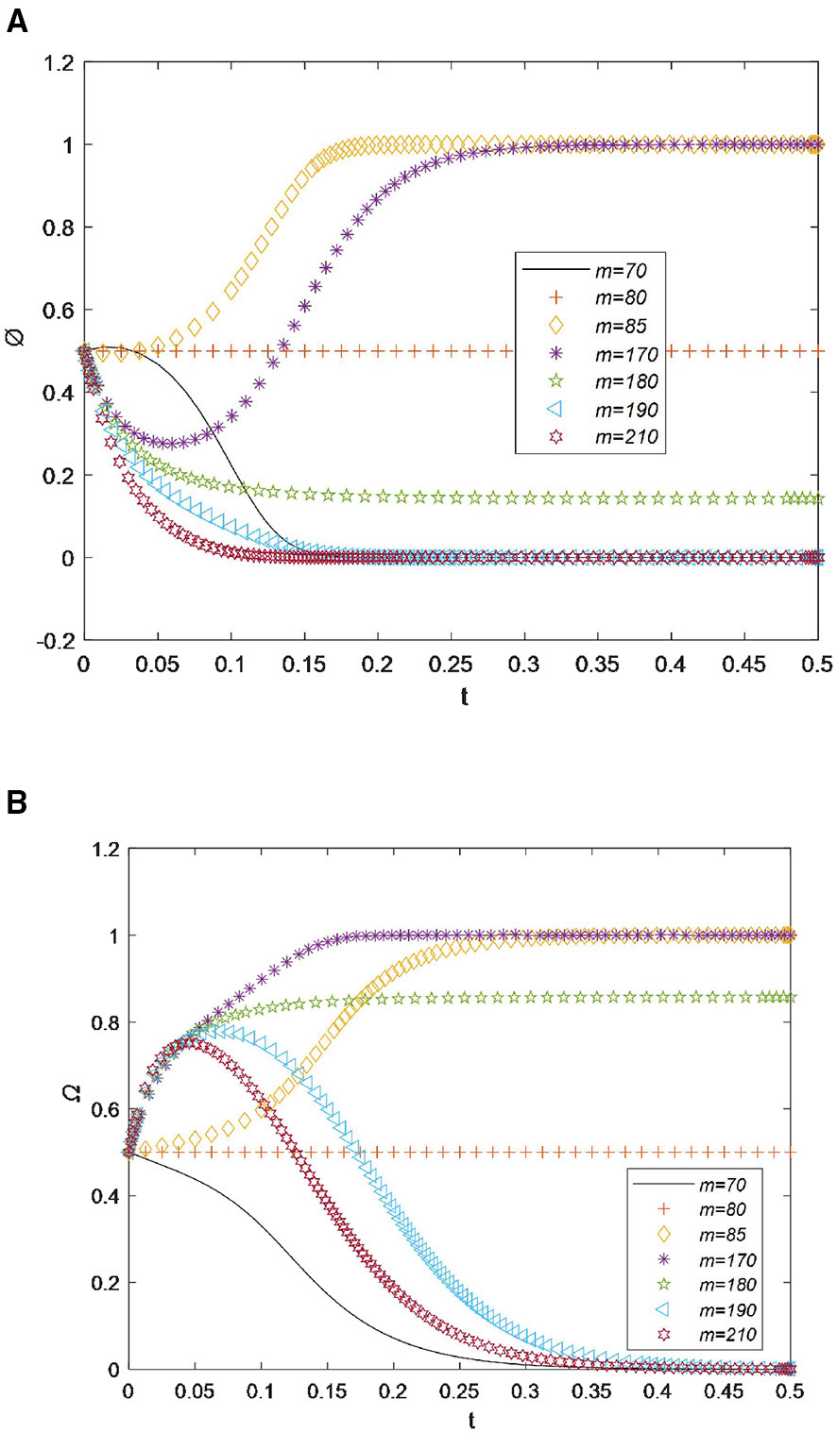
**(A)** Sensitivity analysis of the reward output for drugstores. **(B)** Sensitivity analysis of the reward output for residents.

For drugstores, as shown in Figure 6A, when the reward output *m* equals 70, the willingness to recycle drugs is 0. When it increases to 80, the participation probability rises to 0.5, forming a horizontal line. When *m* increases by only 5, the participation probability reaches its maximum value of 1.0 at the time point 0.15. If *m* continues to increase, the participation probability remains unchanged, but the speed of participation decreases. When *m* increases from 170 to 180, the participation probability rapidly decreases from 1.0 to below 0.2. Afterward, as *m* increases further, the willingness of drugstores to recycle drugs drops to a minimum value of 0 before the time point 0.2. This indicates that excessive rewards increase the burden on drugstores, sharply weakening their recycling willingness.

In Figure 6B, it can be seen that when the reward output *m* equals 80, residents' willingness to recycle drugs is 0.5, forming a horizontal line. When *m* increases by only 5, the participation probability reaches its maximum value of 1.0 at the time point 0.28. If *m* continues to increase, the participation probability remains unchanged, but the speed of participation increases. When *m* increases from 170 to 180, the participation probability rapidly decreases from 1.0 to below 0.80. Afterward, as *m* increases further, the willingness to recycle drugs drops to a minimum value of 0 before the time point 0.35. Therefore, based on the results shown in Figures 6A, B, we can draw the conclusion that drugstores and residents are willing to cooperate and jointly recycle drugs only when the reward output is less than 170 and more than 85.

## 6 Conclusion

Unnecessary and expired drugs are widely present in many countries and regions around the world, posing many serious adverse effects on land resources, the natural environment, public health, and economic development. Therefore, it is important to explore how drugstores can collaborate with residents to recycle these drugs. This study applied evolutionary game theory to establish a theoretical model. We analyzed the cooperative evolution trend and stable strategy of the two parties. The results showed that a stable state of full cooperation between the two participants can be achieved.

This article explores the driving and hindering factors that affect the willingness and depth of cooperation between drugstores and residents. Numerical simulation and sensitivity analysis indicated that socioeconomic performance and moderate reward outputs are driving factors. On the contrary, recycling costs, time loss, promotional costs, large rewards, and small rewards are hindering factors.

We have provided the main ranges of values for these factors based on the numerical settings used in this study. For driving factors, it is recommended that socioeconomic performance be greater than 620 and that the reward output be less than 170 and more than 85. For hindering factors, it is suggested that recycling costs be less than 200, promotional costs be less than 120, and time loss be less than 70.

In terms of policy recommendations, it is first suggested that the government enhance publicity and education to improve residents' knowledge of expired drugs and provide multiple channels for disseminating information on drug recycling, enabling residents to access this knowledge more conveniently and efficiently. Therefore, residents influenced by these measures will become more aware of environmental protection and more attentive to the value of rewards, thereby actively cooperating in recycling efforts. Second, it is recommended that drugstores moderately control the intensity of the reward output. Research has shown that moderate rewards are effective in motivating participation, while excessive rewards can increase the financial burden on businesses. Third, drugstores can disseminate information through WeChat official accounts, corporate websites, and other channels to reduce promotional costs. Moreover, it is recommended that drugstores actively improve operational efficiency, for example, by adopting advanced management methods and novel recycling technologies, to reduce the costs of drug recycling. Finally, it is suggested that residents enhance their environmental protection awareness and personal values. In addition, residents can become more familiar with drug recycling points and channels, thereby reducing the time costs associated with participating in drug recycling.

The evolutionary game model and replicator dynamic equations presented in this article are, to a certain extent, universal and can be used to study similar problems in other countries and regions. In the actual research process, the evolutionary game model can be used after making some minor modifications to some parameters and related formulas according to the specific conditions of different countries and regions.

There are still some limitations in the current study. For example, the investigation of the effects of typical factors is incomplete, and the evaluation of key parameters is not comprehensive. Developing a more complex model, incorporating additional parameters and more intricate coupling mechanisms, may be a valuable direction for future research.

## Data Availability

The original contributions presented in the study are included in the article/supplementary material, further inquiries can be directed to the corresponding authors.
